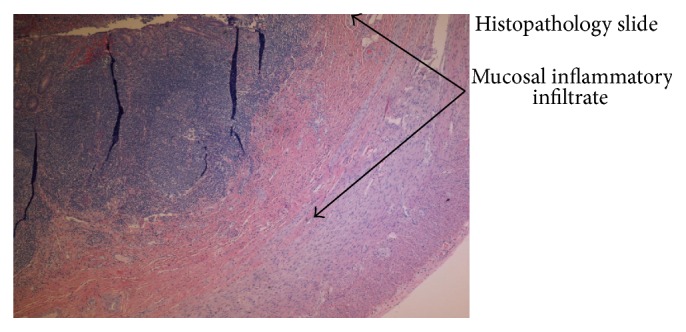# Abdominal Pain in the Female Patient: A Case of Concurrent Acute Appendicitis and Ruptured Endometrioma

**DOI:** 10.1155/2016/2156148

**Published:** 2016-12-20

**Authors:** Martine A. Louis, Amanda R. Doubleday, Elizabeth Lin, Ji Yoon Baek, Alda Andoni, Xiao Hui Wang

**Affiliations:** ^1^Department of Surgery, Flushing Hospital, Flushing, NY, USA; ^2^St. George's University School of Medicine, True Blue, Grenada; ^3^Department of Obstetrics and Gynecology, Flushing Hospital, Flushing, NY, USA

## Abstract

General surgeons are often asked to evaluate acute abdominal pain which has an expanded differential diagnosis in women of childbearing age. Acute appendicitis accounts for many surgical emergencies as a common cause of nongynecologic pelvic pain. In some rare instances, acute appendicitis has been shown to occur simultaneously with a variety of gynecologic diseases. We report a case of concurrent acute appendicitis and ruptured ovarian endometrioma.

## 1. Introduction

General surgeons are often asked to evaluate acute abdominal pain; however the differential diagnosis must be expanded in women of childbearing age. Acute appendicitis accounts for 27.5% of abdominal surgical emergencies and is the most common cause of nongynecologic pelvic pain [1]. In some rare instances, acute appendicitis has been shown to occur simultaneously with a variety of gynecologic diseases, which can add to the diagnostic dilemma. We report a case of concurrent acute appendicitis and ruptured ovarian endometrioma.

## 2. Case Presentation

**Figure 1:**
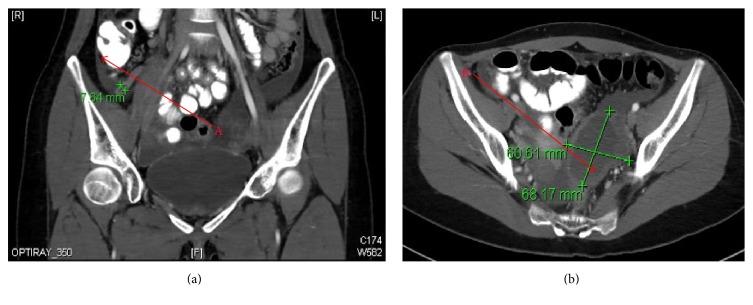
CT scan. A: inflamed appendix; B: left adnexal mass.

**Figure 2:**
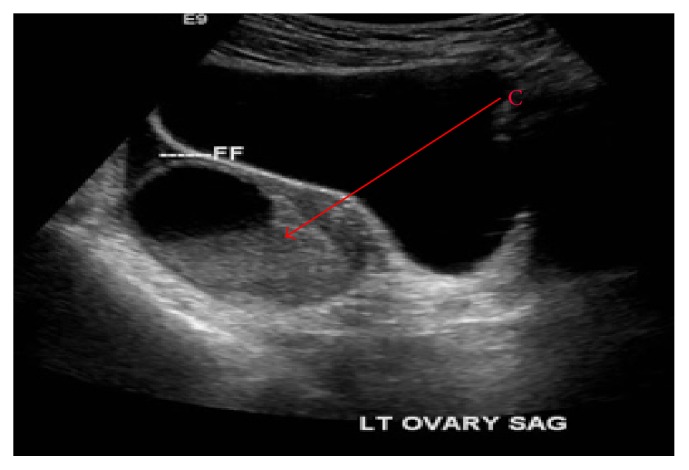
US. C: adnexal mass.

A 24-year-old Chinese woman with no past medical or surgical history was admitted to Flushing Hospital with a one-day history of periumbilical abdominal pain, which migrated to the right lower quadrant. A review of systems was positive for nausea, vomiting, and anorexia, negative for subjective fever or chills. The patient was afebrile and hemodynamically stable. Her physical exam was pertinent for right lower quadrant and suprapubic tenderness, negative for distension, guarding, rebound, or rigidity. Her gynecologic exam was negative for cervical motion and uterine or adnexal tenderness, and her last menstrual period was 19 days previously. Medications included oral contraception. Labs revealed a white blood cell count of 15.9 K/*μ*L with 80.7% neutrophils. Her urinalysis was normal, and her urine pregnancy test was negative. Computed tomography (CT) scan of abdomen and pelvis with intravenous and oral contrast revealed a mildly dilated appendix, measuring 7.5 mm in diameter, and mild wall thickening consistent with acute appendicitis (Figure 1). Additional findings included a large partially cystic complex left adnexal mass measuring 5 × 8.5 × 6 cm with moderate pelvic ascites, which was confirmed by transabdominal and transvaginal ultrasounds (US) (Figure 2). Arterial flow was demonstrated in both ovaries ruling out ovarian torsion.

**Figure 3:**
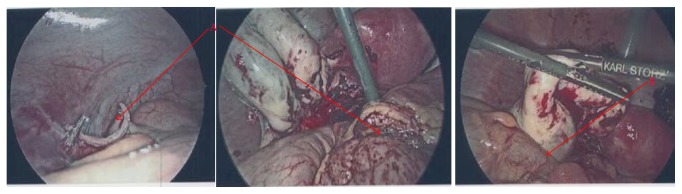
Intraoperative images. A: inflamed appendix; B: ruptured endometrial cyst.

The differential diagnosis included ovarian cyst with secondary periappendiceal inflammation versus acute appendicitis with concurrent large complex ruptured ovarian cyst. The surgical and gynecologic teams, together, performed diagnostic laparoscopy. Intraoperatively, the appendix appeared inflamed, without signs of necrosis, perforation, or abscess formation. The ovarian cyst measured approximately 7 cm and was found to be partially ruptured with dark blood in the pelvis as well as scattered endometriosis-like lesions in the pelvic peritoneum. Laparoscopic appendectomy and left ovarian cystectomy were performed without complication (Figure 3). The pathology report revealed (1) acute appendicitis with appendix measuring 4.5 × 0.6 cm with tan smooth serosa and scant fibrous adhesions and (2) endometrioma measuring 5.5 × 3 × 2.2 cm with tan, gray, purple, rubbery, membranous tissue, without solid areas or papillary tissue. The postoperative course was uneventful with the exception of acute anemia requiring one transfusion of packed red blood cells. The patient was doing well at two weeks postoperative follow-up.

## 3. Discussion

Abdominal pain in young women can present as a unique diagnostic dilemma. A thorough history and physical examination including a gynecologic examination is key to determining the etiology of pain. The differential diagnosis includes but is not limited to acute appendicitis versus gynecologic entities such as ectopic pregnancy, endometriosis, ovarian torsion, or pelvis inflammatory disease to name a few, keeping in mind that they may be concurrent with acute appendicitis or other surgical diseases.

Acute appendicitis carries a lifetime risk of 7% with a peak occurrence between the ages of 10 and 30 years and accounts for 27.5% of surgical emergencies [1]. The annual incidence in the United States is 130,000 per year. Right lower quadrant pain is the single most powerful indicator of acute appendicitis, despite initial periumbilical location, but this may vary with the anatomy of the patient, age, and pregnancy. Anorexia, nausea (90%), and vomiting (75%) are also present. Physical exam likely reveals right lower quadrant tenderness at McBurney's point, localized tenderness to percussion, guarding, positive psoas sign, obturator sign, Rovsing's sign, and/or Dunphy's sign. Leukocytosis (>10000 per mm^3^) is present in 80% of cases; however, leukocytosis alone has a low specificity. An elevated WBC count in conjunction with neutrophilia and elevated C-reactive protein level, however, has a sensitivity of 97 to 100%. Ultrasound (sensitivity: 83%) with a noncompressible appendix > 6 cm diameter and CT scans (sensitivity: 90%) identifying periappendiceal inflammatory changes are used to aid in diagnosis [2]. On histopathology, acute appendicitis is characterized by mucosal ulceration and transmural polymorph infiltrate often with mural necrosis and a serosal inflammatory response which is demonstrated in the pathology slide of our patient's appendix (Figure 4) [3].

Endometriomas, also known as chocolate cysts or endometrioid cysts, contain dark reddish, brown degenerated blood products. They occur during reproductive years in 17–44% of all women with endometriosis. Infertility and dysmenorrhea occur as the cysts are most often found in the ovaries and less commonly in anterior/posterior cul-de-sac, the uterosacral ligaments, uterus, or colon. Physical exam findings include tenderness or nodules in the cul-de-sac or uterosacral ligaments, pain with uterine movement, enlarged adnexal masses, or fixation of adnexa or uterus in a retroverted position. Plain X-rays can show calcified endometrioma 10% of the time. Transvaginal US, CT, and magnetic resonance imaging (MRI) can aid in diagnosis. On US, 50% classically appear as a unilocular cyst and less commonly a multiloculated cyst, a cystic-solid lesion (15%), purely solid lesion (1%), or anechoic cyst (2%) in postmenopausal patients [4]. Findings on CT scans show a hyperdense focus inside an ovarian cyst that may help differentiate an endometrioma from other pelvic masses. On MRI, well-defined markedly hypointense foci with the cystic lesion on T2-weighted image had a sensitivity of 93%, but low specificity of 45% as other types of hemorrhagic cystic adnexal lesions, such as functional hemorrhagic ovarian cysts, appear similar [5].

Ectopic pregnancy can occur in the fallopian tube, cervix, ovary, or abdomen with an incidence of 60,000 per year in the United States [6]. Rupture of ectopic pregnancy is a medical emergency and may result in significant abdominal or pelvic pain. In females of reproductive age, urinary or serum level of *β*-human chorionic gonadotropin (*β*-hCG) and pelvic US aid in the diagnosis.

Pelvic inflammatory disease (PID) may lead to endometritis, salpingitis, oophoritis, peritonitis, perihepatitis, and tubo-ovarian abscess. PID has an incidence of 1,000,000 women per year in the United States. Common findings include vaginal discharge, urinary symptoms, history of PID, tenderness outside the right lower quadrant, cervical motion tenderness, and positive urinalysis [7]. Pelvic sonogram and CT scan identify fat stranding, endometrial fluid, debris, and indistinct tissue planes [8].

Hemorrhagic corpus luteal cysts commonly occur in women of childbearing age between days 20–26 of the menstrual cycle or during the first trimester of pregnancy, accounting for hospital admissions in 4% of women before age of 65. Ruptured cysts are associated with sudden onset of unilateral lower abdominal pain, nausea and vomiting, vaginal bleeding, weakness, syncope, and shoulder tenderness. Ultrasound, CT, and MRI may identify the hemorrhagic ovarian cyst or an adnexal mass associated with high attenuation pelvic fluid representing hemoperitoneum [9].

Ovarian torsion occurs when ovarian cysts or neoplasms, usually ≥5 cm, twist around the vascular pedicle compromising blood flow and resulting in ischemia of the ovary, which can lead to necrosis, hemorrhage, or peritonitis [10]. Ovarian torsion has been estimated to account for 2.7% of surgical emergencies. Physical exam may reveal a palpable adnexal mass [11]. Color Doppler sonography identifies compromised blood flow, and the treatment is always surgical detorsion.

Appendiceal endometriosis occurs as a separate entity with an incidence between 0.054% and 0.8% [12]. It may present with symptoms similar to acute appendicitis, endometriosis, melena, lower gastrointestinal bleeding, cecal intussusception, and perforation [13]. Some patients have been found to have concomitant appendiceal pathology [14]. Although there are no current guidelines for appendectomy in patients with endometriosis, appendectomy may be considered as part of surgical treatment in endometriosis to avoid misdiagnosis in the future.

## 4. Conclusion

This case illustrates how concomitant gynecologic disorders can occur in patients with acute appendicitis. Our patient presented with an acute onset of epigastric pain that radiated to the right lower quadrant, tenderness at McBurney's point, and leukocytosis which are classic findings in acute appendicitis. However, her low hematocrit and suprapubic pain were atypical. Interestingly, she had no cervical motion tenderness or palpable adnexal mass. Imaging reported acute appendicitis and endometrioma. This serves as a reminder that an atypical clinical picture in women should also promote imaging studies with a goal of increasing diagnostic accuracy. We were able to appropriately treat this patient with a multidisciplinary approach. Such an approach can help to prevent system failures and improve patient safety and outcomes.

## Figures and Tables

**Figure 4:**